# Book Reviews

**Published:** 1828-11

**Authors:** 


### CRITICAL ANALYSES.

Quæ laudanda forent, et quæ culpanda, vicissim

Illa, prius, cretâ; mox hæc, carbone, *notamus*.—PERSIUS.

*A Treatise on Gout, Apoplexy, Paralysis, and Disorders of the Nervous System.* By A. RENNIE, Surgeon, &c.—8vo. pp. 213. Burgess and Hill, London, 1828.

ALTHOUGH gout has been a constant subject of study and investigation ever since the days of HIPPOCRATES, and early attracted observation and research, by evincing singular hostility to the great and the learned; yet, in spite of every barrier which medical talent and industry have ever been able to suggest and raise in opposition to its prevailing ravages, it has hitherto, with its single but hydra ally, civilization, scorned the control of physic, and extended its tyranny from the mansion to the hovel,—from the inactive limbs of the scholar, to those of the laborious and unlettered mechanic; till it has at length achieved a monopoly, and deprived its martyrs of the petty consolation of being connected, in sufferings at least, with men of rank and genius.

We therefore contemplate with pleasure every new endeavour which is made to erase this formidable disease from the list of our *opprobria*, and rescue mankind from its severe and prevailing oppression.

Mr. RENNIE is not only amongst the few who institute their inquiries into the arcana of gout upon sound practical principles—the examination of facts, but he carries his views back to the earliest dawn of its existence, and endeavours to distinguish and point out its characteristic features in the embriotic and imperfect state of its peculiar diathesis. And he justly remarks, that

" Due distinctions have not been made and preserved betwixt what may be termed the gouty diathesis, disposition, or tendency, and the actual disorder of the fit. Whereas, these states of constitution are essentially different, and ought to be marked by a broad line of demarcation. The causes which produce the gouty disposition are generally very distinct from those which excite the fit. The symptoms indicating the mere disposition to gout are also very different from those which occur when the disease is developed. The methods of curing the two states of disorder are still more widely different, and sometimes directly opposite. An invalid may possess all the peculiarities of the gouty disposition, from habitual exposure to the disposing causes of the disease, and yet for months or years entirely escape any actual fit. Those who are subject frequently to attacks enjoy intervals of freedom, in which no decidedly gouty symptom is felt: still they possess the diathesis, and that sometimes so strongly that the slightest exciting cause will induce the fit. He who possesses the gouty disposition merely, and is anxious to get cured of it, must pursue a very different course from him who actually labours under a fit, and wishes to be cured of that: he must not only prevent the fit, to which he might be liable, by avoiding the exciting causes; but he must farther adopt means calculated to restore that morbid peculiarity of constitution which renders him subject to the specific disorder called gout. This he can hope to accomplish only by avoiding all the disposing causes of the disease,—by counteracting their effects where they are unavoidable,—and by restoring the constitution when it has already suffered from their operation. I believe it will be found that it is from want of due attention to these important distinctions that so many different views have been entertained of the nature of gout, and so many contradictory methods of treatment have been resorted to. Most of these may have been occasionally serviceable, but not one has been found generally or permanently successful; and for very obvious reasons: they have not been suggested by any clear, sound, and comprehensive acquaintance with the true causes and nature of the disease, but simply by a desire to combat its more prominent and severe symptoms. Partial or occasional relief in this way, obtained for the most part at first by mere accident, has gained to certain remedies an unmerited popularity, and has led even medical practitioners rather to confide in the limited, deceptive, and uncertain experience of occasional cases of success, than to inquire into and establish the principles on which this or that remedy has produced its results.

" The way to discover a safe and effectual method of curing any disease is surely not to go hunting after fancied specifics, applying them in every fresh case that occurs, with a blind and uncertain temerity. This, in fact, is nothing else than to make the human constitution a subject of empirical experiments; and, if ever disease has given scope for experimenting with rash and hazardous remedies, the gout is that disease. What with rules of abstinence and starvation, cold applications, the Portland powder and other indiscriminate tonics, the eau medicinale, colchicum, and such other deleterious narcotics, producing temporary relief, but real and irretrievable injury,—it may be truly said that remedial measures, indiscreetly used for the cure of gout, have shortened more lives than the disease itself would have done. Common sense, and the sad experience of numberless unfortunate cases, therefore, alike admonish us no longer to proceed groping our way in the darkness of empiricism, but to go at once to the root of the matter, under the conviction that the true method of arriving at a cure for the malady is, in the first instance, to ascertain what are its real causes; and, secondly, to endeavour to demonstrate its real nature. This once accomplished, if experience has shown any methods of treatment to be beneficial, we are thus enabled to judge on what principles these beneficial effects have been produced, and we are prepared to apply them with success,—at all events with entire safety in new cases.

"In conducting this inquiry into the causes and nature of gout, we shall draw an accurate and broad distinction betwixt the mere disposition or tendency to gout, and the disease itself; between the causes which induce the gouty disposition, and those which excite the paroxysm in its peculiar and truly distressing symptoms." (P. 3.)

He then delineates the general arrangement of his subject, of which we quote the first division, the development of which alone occupies the present volume.

"The Gouty Habit or Diathesis.—That peculiar habit or state of the body induced by the disposing causes, and constituting the liability to a paroxysm or fit of gout, from exposure to the occasional exciting causes.

"Here we shall inquire into—

"1. The history of the circumstances, constitutional or extraneous, which generally attend the origin and progress of the gouty disposition.

"2. Present an historical description of those symptoms by which the gouty habit of body is indicated.

"3. Inquire into the true causes usually concerned in producing the tendency to gout.

"4. From the facts adduced we shall draw certain pathological conclusions, tending to show what is the peculiar nature of the gouty habit of constitution; what disordered state of the functions of the body constitutes the gouty peculiarity." (P. 7.)

The author's history of the circumstances, &c. is unnecessarily long and verbose: it comprehends thirty-two distinct general observations. Amongst the predisposing circumstances, he admits that "the children of gouty parents are, upon the whole, more frequently subject to this disease than others;" but also affirms that the children of those who have fallen victims to apoplexy and paralysis are extremely subject to gout.

He considers the *male sex* more liable to regular acute gout than the female, but contends that this opinion is carried farther than facts will warrant; and asserts, what few will deny, that gouty affections, of an irregular and internal description, are very prevalent among women.

Though SYDENHAM, CULLEN, SCUDAMORE, and others, affirm that people of a robust make and constitution are peculiarly exposed to gout, yet, since "all impartial observers" own that it not unfrequently occurs in those of a contrary make, and Sydenham himself says that "sometimes, though seldom, it seizes thin folks," therefore we are fairly prevented from concluding that gout arises from plethora.

The author lays great stress on the facts that its victims are not the young and vigorous, but those worn by age, disease, excesses, &c. and such as are placed in circumstances tending to exhaust the nervous energy : and in this way he considers excessive indulgence in venery a powerful promoter of the gouty tendency or diathesis.

But, though indolence and habits of feasting, and a too-nutritious and copious table, have been generally set down as principal causes of gout, still it is equally true that many temperate individuals,—some real disciples of CORNARO,—and those who suddenly exchange luxury and a full diet for abstemiousness and a meagre fare,—and others, on the contrary, who emerge unexpectedly from poverty and starvation to affluence and the enjoyment of sumptuous repasts, are all occasionally seized with gout, notwithstanding their various and even opposite modes of living. Mr. Rennie, therefore, here is justified in affirming, that "no view of the causes and nature of the disease can be correct which does not reconcile in a satisfactory manner these very opposite facts."

"Gout has been observed to be much more prevalent in certain classes of society than in others. This evidently arises from the particular pursuits, pleasures, habits of diet and regimen, and various external circumstances incident to different stations in life." (P. 10.)

"Although gout undoubtedly is more frequent amongst the affluent and higher circles, it is far from being confined to such classes. In some form or other, it may be found from the palace to the cottage. Whence the far-famed distinction, the *poor* gout and the *rich*.

"The gout of the humble walks most frequently assumes the irregular, anomalous, and internal forms, and is often complicated with other diseases. Genuine gout, in its decided, severe, and intractable forms, prevails chiefly amongst the higher and opulent ranks. It merits notice that, in certain circumstances of climate, for instance in the northern latitudes, the disease is almost exclusively confined to the rich. In the cyder districts of England, the poorer orders are very generally afflicted with the malady.

"The servants of the rich, who enjoy in ease and repletion the advantages of wealth, and are subject in no small degree to its temptations, often become gouty. The butler and coachman are often seized, or a favorite pampered valet. Also publicans, and the drivers and guards of mails and coaches, as I am informed." (P. 11.)

The author then proceeds to enumerate a variety of circumstances, physical, moral, and intellectual, all of which, by general consent, predispose more or less to nervous debility; and these he considers proportionably powerful in producing a tendency to the disease of which he treats.

In his remarks on climate, Mr. R. asserts that

"The inhabitants of insular situations, and especially on the borders of low, damp, level districts, subject to agues, are much disposed to gouty affections. The climate of England possesses supereminently those very peculiarities which general observation has shown most productive of gouty disorders. Accordingly in England gout abounds amongst all classes. Take a damp, swampy, variable climate, everywhere within the greater extremes, and you find gouty disorders also abound." (P. 13.)

But the fact that gout is little known in Holland would seem to indicate that the author attaches an exaggerated influence to climate. VAN SWIETEN, it is true, attributed the exemption which his countrymen enjoyed from gout, not to the salubrity of their climate, but to their ignorance of the use, or rather the abuse, of the vine.

After stating that one of the most frequent and invariable forms of disordered circulation to which the gouty are subject is a determination of blood to the head, the author makes the following interesting observations, in which he most unequivocally displays his opinion as to the nature of gout.

"In persons whose constitutional energies have been much impaired, this takes place sometimes in a very direct and unexpected manner; but determination to the head much more frequently ensues as a consequence of the previous disordered states of the circulation just noticed. Persons liable to pulmonary disorders, to obstructed liver, to dyspeptic stomach, and a congestive obstructed state of the bowels, have on all occasions shown a peculiar tendency to the farther complication of determination to the head previous to the accession of gout.

"'It is well known,' says Dr. Parry, 'that the diseases which more especially precede gouty paroxysms, or occur in their intervals, are those of the alimentary canal and head.' And he endeavours farther to show that all those symptoms usually attending gout, and styled nervous, 'proceed mainly from a determination of blood to the alimentary canal and head.'

"While we coincide with Dr. Parry in the observation of the fact as here stated, we can see no grounds for banishing the nervous system from all pathological inquiries, as that author has done. However difficult it may be to separate the functions of the vascular system from those of the nervous, which no one can ever expect to do with any success, there still remains a most important class of disordering causes, which produce their effects on the body chiefly, if not solely, through the medium of the nervous functions; and the particular states of the nervous system, and the laws regulating its functions, must always constitute an important branch of pathological inquiry. In tracing gout to its remote causes, the condition of the nervous system demands a peculiarly close attention.

"Many, if not all, of the causes of gout produce their effects chiefly through the medium of the nervous system. In viewing determination of blood to the head as one of the principal and invariable of the causes which induce gout by disordering the nervous system, we may be near the truth; but it is not the only originating cause of this malady; numerous others might be referred to as lending their influence." (P. 20.)

We think our author speaks too sweepingly when he maintains "few there are who do not become remarkably *nervous* previous to falling into gout." We are ready to admit that few individuals are long visited by gouty paroxysms who do not become more or less the subjects of morbid feelings and a depressed mind; but we incline to believe that thousands receive their first visitation with a mind which has till then continued undisturbed, and habitually free from the intrusion of anxiety or imaginary fears and conceits.

In his history of the symptoms which characterise the gouty habit, the author scarcely omits the enumeration of any which, directly or indirectly, might be supposed to indicate a loss of nervous energy, either in the entire system or in some of its most important organs. And we think this part of his work will be considered unnecessarily minute and redundant. His observations, however, for the most part, are practical and judicious, and, barring two defects, those of repetition and amplification, might pass uncensured. Yet the commission of these faults is so destructive to the comfort and instruction of the reader, and the omission of them so easy for the writer, that they deserve severe reprehension in all professional works; in which, nevertheless, they most abound.

"The origin of the gouty disposition is usually slow and gradual, and sometimes imperceptible to the individual himself. The symptoms by which its advance is indicated vary considerably in individual cases. This is only what might be expected from the variety of circumstances just detailed, under which this habit is acquired. And not only are the external circumstances various and dissimilar, but constitutional peculiarities of the most diverse nature occasionally terminate in gout, under exposure to certain causes or combination of causes.

"One man is powerfully muscular, another feeble and slender; one is corpulent and unwieldy, another spare and agile; one is a perfect specimen of conviviality, given to every luxury and excess,

—another a pattern of sobriety, temperance, or starvation; one is of habits unceasingly active, while he has the power of motion,—another is by necessity, or profession, or pure laziness, addicted to indolent inactivity; one is of a sanguine, energetic temperament,—another nervous and irritable,—a third phlegmatic,—a fourth melancholic; one is full, florid, and plethoric,—another pale, sallow, papery, has been subjected to bleedings and cuppings without end, till he has perhaps lost and renewed every particle of red blood in his body. We have one habitually dyspeptic for years,—another continually bilious,—a third naturally (i. e. constantly) costive,—another quite the reverse. One has been subject to acute inflammations,—others to long-continued organic affections; while some few have never known what disease of any kind was, but have been accustomed to laugh at physicians, and to enjoy with seeming impunity every luxury and indulgence that might fall in their way. Yet one and all of these, somehow or other, find themselves, at some period in their passage through life, arrested by this unwelcome malady.

"Seeing the circumstances and constitutions of the gouty are so diversified and so opposite, and that the causes producing the diathesis are so various in kind, degree, and combination of influence, it is plain that no description, however minute, could be found to embrace the symptoms peculiar to every case." (P. 23.)

After stating that muscular debility generally precedes gout, and evinces its existence in the muscular coats of the intestines by their excessive distention with flatus, and in the abdominal muscles by a soft and flabby state of the belly, which becomes "in the corpulent loose and pendulous," he maintains that the corpulency is seldom real, and adds—

"Fulness and enlargement of the abdomen is by no means necessarily attendant on the gouty state; for sometimes the belly is so small in certain enervated, emaciated invalids, that you can feel the aortal pulsations almost under your finger, the abdominal parietes lying close on the spine; as happened lately in a young man, who had lain a month in bed in excruciating tortures before I saw him, and who was never accustomed to full living, who was spare, pallid, and the very reverse of plethoric, (*uxore sterili permasculâ,*) and he had been on low diet for weeks. This case was put under medical regimen, and ordered animal food thrice daily, with wine, and in a week or two he was walking about entirely free from gout. How absurd then to tell us that gout proceeds from plethora!" (P. 26.)

The author then depicts the various alterations in the locomotive powers, and the divers signs by which the form and countenance proclaim the commencement of a gouty tendency in the constitution. "The derangement of the circulation,"—"the complexion characteristic of the gouty state,"

—"the state of the skin,"—the functional disturbances likewise arising from disordered distribution of the blood in gouty cases, are all expatiated upon; and the different train of phenomena as they occur in pulmonary or hepatic congestion described.

"Another indication of approaching gout is a more than usual tenderness and delicacy of constitution, and a greater susceptibility to be injured by the disposing causes of the disease.

"This delicacy of constitution gradually increases more and more, so that the invalid, whatever he may have been in former times of health and vigor, now feels himself, earlier than is natural to his age, sensible of growing infirmities. In fact, a 'premature old age,' as Sydenham terms it, comes on, in direct proportion to the expenditure of the constitutional strength, by those imprudent excesses in which he has indulged; and, before the full extent of the mischief is known, it is generally too late to prevent the consequences.

"The constitution thus impaired becomes more and more susceptible to climate and weather, especially feeling the effects of cold, of wet rainy weather, and of sudden changes; also of easterly winds, of evening air, and the access of winter. He finds it necessary to be more attentive than formerly to his dress, which he must carefully adapt to the weather. Indeed, he is continually subject to colds and rheumatic affections on the slightest exposure.

"The approach of November, and the wet and cold of winter, brings along with it the certainty of some serious attack of illness, which on each returning year requires more care and confinement to restore. The severity of the winters, and the moisture and changeableness of an English climate, are now a constant source of anxiety, the never-ceasing theme of complaint! Add to this the necessity of remaining within doors in unfavorable weather, in which case he suffers greatly from want of exercise, especially if in a close tainted atmosphere; becoming languid, nervous, irritable, flatulent, dyspeptic, melancholy, sleepless, and despondent. He is much more easily fatigued than he ever before recollects, whether by corporeal exertions or even mental efforts, which are a severe exhausting drudgery, and he naturally prefers the lightest and most frivolous amusements. Wakefulness, or late hours in company, or otherwise, soon show their effects on his countenance, in the pale, haggard, worn-out aspect, and especially if attended with too much wine. Hard study, close reading, or precise calculations, produce much confusion of mind, languor, and irritability; in fact, he is hardly capable of fixing his attention. Unusual abstinence or excess are alike hurtful. Confinement of the bowels or flatulence, and in fact the slightest derangements of the digestive functions, are attended with much annoyance.

"He has of late become so delicate, that if he changes his bed, or shifts his flannels, or gets damp feet, or is exposed out of doors in wet chill weather, or in easterly winds; or if he has dined out, and taken a glass too much, or has not been very circumspect in what he eats, he is sure to suffer a degree of inconvenience to which he was in former periods a stranger. He tosses half the night in sleepless anxiety and disquietude; and dismal dreams, pregnant with confusion and horror, infest his broken slumbers. In short, a thousand undefinable sources of continual and serious inconvenience now attract his attention, which he never discovered before; and if, regardless or unaware of their hurtful influence, he continues exposed to it, a long train of new and uneasy symptoms prey upon his comfort. No wonder that he is depressed and despondent. Those who have had any of the important organs habitually disordered, are subject to attacks of serious disease." (P. 28.)

Then indigestion, with its host of distressing satellites, are brought into prominent notice, and treated with all that minute attention which they have been accustomed to receive from most writers on this disease.

"There are however invalids, verging upon the gouty habit, or already fallen into it, who, from long-continued dyspepsia, have learnt the necessity of consulting their stomach in every article of food they put in their lips: they practise self-denial, and discipline their taste most cautiously, for the sake of their weakened stomach; and, by dint of long experience, they do come to discover what food agrees with their digestion. But when their stomach, from the simple state of dyspepsia, has undergone the farther transition into the gouty peculiarity, these invalids will find all their former experience quite at fault. They have been accustomed to judge of suitable diet chiefly by their sensations of comfort at stomach after meals. Now this criterion fails them entirely, and they may take, from day to day, the most hurtful diet, without ever being aware, from the sensations at stomach, that it is hurtful. Woefully are they chastised for their unconscious errors by the invasion of gout, to them quite unaccountable. Such invalids as these can no longer trust to their own experience. To them a new set of rules is necessary, rules founded on sound views of the true nature of gout, aided by a correct knowledge of their peculiar constitution.

"This tendency of the stomach to be disordered by food which creates no uneasy sensations at the time, is a very curious circumstance; and is an almost invariable attendant of that weakened condition of the organ characteristic of gout.

"Imperfect digestion of fat is often met with in gouty stomachs, and the smallest particle will disagree. Butter and pastry also lie sour and rancid on such stomachs. Cheese also, salads, and cold or acid fruits and acid liquors, are uniformly hurtful, occasioning effects quite specific on such stomachs." (P. 46.)

The various states of the bowels, which usually mark, according to Mr. Rennie, in legible characters the gouty tendency, he next enumerates with much minuteness and care. After stating that costiveness is a common forerunner of gout, and that its effects have for centuries been incorrectly attributed by physicians to plethora, which opinion is contradicted and annulled by the fact that bleeding will sometimes, in such cases, superinduce a paroxysm of gout; to effect which, starvation has a still greater tendency. He adds—

" *Periodical purgings.*—Besides habitual costiveness as characterising the gouty habit, there is another state of the bowels to which they are sometimes subject, quite the opposite, and that is periodical purgings.

" It is a very curious observation, that those people who have been most remarkable for general costiveness for years before acquiring the gouty habit, will become subject to sudden attacks of purging shortly previous to the accession of gout. These spontaneous purgings are to them a new, and altogether unaccountable, change in their habit. They are at first charmed with the relief so produced, and hail it as a salutary crisis of nature. Farther experience, however, generally convinces them of the contrary. They come to find that their bowels have acquired an extraordinary degree of irritability, that they are weakened in tone, and remarkably under the influence of the weather and seasons; and the slightest errors in diet affect them with disturbance. Those sudden purgings become occasionally violent and protracted, with severe twisting pain, straining at stool, and great general debility; and, after the purging subsides, there is not felt that happy relief at first anticipated, but a degree of flatulence at stomach, indigestion, perhaps obstinate costiveness, frequent desire to go to stool without any effect, bearing down efforts, and perhaps piles.

" There are various kinds of diarrhœa to which gouty habits are subject.

" 1. Simple watery diarrhœa. This occurs usually in debilitated habits, after any exposure to cold or wet while the body is heated; chiefly in spring and autumn. It is at times very severe and protracted. I have known an attack last for three or four weeks almost uninterruptedly, causing much emaciation and general debility. More usually, however, an attack lasts three or four days; after which, a relapse to obstinate costiveness ensues, with tendency to piles, uncomfortable fulness in the stomach, uneasy sensations in the head, and sometimes gout.

" 2. Bilious diarrhœa. When costiveness has continued some time, bilious diarrhœa sometimes operates as a spontaneous relief to the disturbance thereby occasioned. The bile, long pent up in the first passages, gets exit into the alimentary canal; irritates the sensible mucous membrane by its acrid stimulus, with twisting, griping pains, and rolling sensations in the bowels, which create great depression. The bile is at length discharged in copious quantities, acrid and hot, and often excoriating the anus, causing very violent and painful evacuating efforts.

"3. In some cases, especially in autumn, these biliary purgings are still more severe, and assume all the peculiarities of cholera morbus; spasms in the bowels, exquisite griping at the navel, sickness, vomiting, tenesmus, violent pain at stomach and duodenum, which feel as if twisting themselves in all possible contortions; anxiety, cold sweats, and great depression of strength. Such attacks as these usually come on after some exposure to cold and wet, while the body is heated; beginning with chilliness, cold feet, spasms, severe headache, giddiness, &c. Errors in diet contribute. Fruit eaten when the stomach is loaded with bile, or while the perspiration is checked suddenly; also cold drinks will cause the same.

"In some cases of diarrhœa, the stools are mixed with blood; and periodical purgings of blood are not uncommon in gouty people.

"4. There is another affection of the same kind to which gouty habits are sometimes subject, especially such as are much reduced and disposed to dropsy, and who reside in fenny aguish districts.

"It begins with chilliness and shivering, cold extremities, great depression and sense of sinking, often swimming in the head, and sense of great weight and pain across the loins, and betwixt the shoulders; there is sickness and oppression at stomach. The invalid is obliged to go to bed. Then succeed reactive fever, wakefulness, mental wandering, nervous confusion, spasms, severe pain in the bowels, and purging. The stomach and bowels are enormously distended with pent-up flatulence, with a rumbling noise and twisting; irritative, griping action around the navel and in the colon. There is painful tenesmus, frequent watery stools, with much straining and blood.

"The skin is cold and clammy, with partial sweats; the pulse quick and hard; tongue coated, but moist. Such an attack I have known continue for weeks, till actively treated, and then pass off in a fit of gout in the extremities.

"It is curious to observe how subject gouty invalids are to cholera and bilious flux, at those seasons when the disorder prevails.

"In these attacks of watery, bilious, or bloody diarrhœa, the bowels seem to be affected with an inordinate sensibility, with a determination of blood to the mucous membrane; and this determination is the consequence of that weakened irritable state of the general habit, produced by the disposing causes." (P. 50.)

He then briefly notices the state of the kidneys and urinary passages; and regarding the peculiarities of the urine, he coincides with SCUDAMORE.

The following are the other heads under which he has assembled a vast catalogue of symptoms, which he considers amongst the characteristics of the gouty diathesis. " Perspiration. A tendency to conversion of various functions into each other: as example, attacks of purging remarkably alternate with copious perspirations. Nervous symptoms. The state of the mind. Sleep. Impaired perception of the senses. The temperature of the body."

He concludes with the ***history of symptoms,*** by saying—

" In general, amongst the gouty who inhabit large towns, where the air is confined and impure, nervous symptoms predominate. In the inhabitants of the country, disorders of the circulation are more prominent; the sanguineous diathesis prevails, and gout partakes more of the inflammatory character. In the habitually intemperate and luxurious, and the debauchee, the digestive organs attract the greatest attention. In all cases disturbance, in a greater or less degree, in these three important orders of the functions, invariably precedes and attends the gouty diathesis. The symptoms never are confined to one alone, although sometimes more particularly prominent in one than in another, according to circumstances. The causes, too, which dispose to gout are all such, in their nature and operation, as to affect each of these functions necessarily and directly; and, when these causes have been in operation, the symptoms which we have described are the natural result. This leads me to treat of the disposing causes of the gout in their operation and effects on the constitution. What these causes are which engender the gouty tendency, and, from a state of soundness and health, bring the constitution into such a state of depression and continual liability to suffering, is an inquiry most interesting to every gouty sufferer, and not less so to the pathologist." (P. 78.)

" ***Inquiry into the true Causes usually concerned in producing a tendency to Gout.***"

The hereditary nature of gout is discussed by the author at considerable length, and he cites many authorities pro and con; but leaves the question undecided, after shewing a preference for the positive side. He will not allow *sex* to have any influence in producing the diathesis; and contends that women are only less liable to gout because less exposed to its causes; that the state of menstruation has no connexion with the disease, since it takes place alike before, during, and after the cessation of the menses,—when they are scanty, copious, or irregular. " There is no uniform rule."

" Have form, size, or proportions of the body any influence in predisposing to gout?"

" When slender and originally delicate persons are seized, we always find some peculiar advantages existing in their favor, calculated to sustain their constitution under the impairing causes to which they are subject. Affluence and the comforts of life will preserve many an invalid from a premature grave, leaving him under a long protracted struggle with the gout; whereas, were he poor and necessitous, and exposed to hardships, he would not have survived. So a strong constitution will enable a man to struggle long with impairing causes under the gouty diathesis, when, if he had been weak, he must have fallen an early sacrifice. This is the true explanation of the size and athletic form of so many whom we find doomed to a gouty old age. It is the large head, capacious chest, and original organic vigor, which has carried them through; and there is no connexion whatever between the size, form, and strength of the body, and the gouty tendency, further than this,—it is merely a coincidence modifying the influence of the other disposing causes; and this is confirmed by considering the question which follows." (P. 87.)

He considers that age has no direct influence in producing this diathesis, since, *cœteris paribus*, those who are exposed to the greater number of debilitating causes are uniformly the earliest victims of gout.

Indolence is not really a cause of gout, but has been mistaken as such, because exercise, which greatly increases the nervous energy, prevents its recurrence from other deleterious influences. Impure air, with Mr. Rennie, has a most preponderating influence in producing gout; which opinion he supports with much good sense. He attributes nearly an equally injurious influence to defective clothing and damp beds.

"The practice of sleeping in bedclothes that have contracted damp is of all others the most prevalent, yet the least appreciated, mode in which various serious disorders are brought on in this country. In a moist climate such as England, particularly in the winter season and rainy weather, this is almost universally the case, and no sufficiency in the building of houses can effectually prevent it. Woollen blankets, in particular, imbibe moisture from the surrounding air with avidity, and are always saturated in proportion to its humidity. This is easily put to the test by holding woollens, that have been in disuse for a day or two in damp weather, to the fire: the heat causes the moisture to evaporate as perceptibly as if damped on purpose. A person who envelopes himself nightly in bedclothes thus charged with moisture can hardly escape evil consequences. Even the most robust, on occasions, contract serious illness from sleeping in a bed more than usually damp. How much more, then, the enervated and susceptible frame? Some such, it is well known, from getting a damp bed, in travelling or otherwise, contract disease from which they never recover. Inflammations, severe rheumatisms, and various chronic disorders of the internal organs, may often be referred to this origin. But there are, besides, large classes in the community who, in a very insidious and imperceptible manner, fall into bad health, and linger for years under chronic maladies, the real origin of which they cannot discover; and such people will find a degree of dampness of their beds, hardly often perceptible to the sensations, to be no unusual source of their sufferings." (P. 115.)

He next examines the influence which excessive study, the passions, copious bleedings, climate, locality, and the seasons, and errors of diet, exert in producing the gouty diathesis, with much logic and emphasis; and apportions to each a degree of power expressed by the ratio in which they individually exhaust the energy of the brain.

The author at length enters on the description of the diseases which dispose to gout. But here we forbear to follow him, as what he relates regarding these is, for the most part, an amplified recapitulation of what was either distinctly said or implied in former parts of the volume.

The following extract, with which we close our remarks, contains a pretty correct outline of the author's doctrines touching gout.

"From the various facts and observations that have been adduced, the inference seems direct and natural, that the gouty peculiarity of constitution depends, directly and essentially, on a certain condition of the brain; and that a correct and clear view of the relations of that organ to the other functions of the body, and a due estimate of the peculiar consequences arising from certain morbid states of the cerebral functions, furnishes the true key to the pathology of gout, hitherto so much an object of anxious research. If this view of the disease, so little dreamed of by those whom this disorder has so perplexed and baffled by its ever-changeable and unaccountable features, could be established on clear and substantial grounds, more would thereby be gained in the progress towards a sound and scientific method of treating the disease, than by all the attempts hitherto made at the discovery of a specific remedy for the malady.

"The position that gout depends on a certain state of the brain, is not a speculative and visionary theory: it is supported by a mass of concurrent evidence, which embraces the whole history of the causes and symptoms of the disease, as well as by the effects of every remedial measure that has been attended with success.

"That state of the brain in which I conceive the essential peculiarity of the gouty diathesis to consist, is, in the first place, a deficiency of the cerebral power; and, secondly, a congestive state of the cerebral vessels, and these two conditions being co-existent.

First, with respect to the cerebral power, we have already shown a variety of very influential agents by which this is impaired; and we have shown, by incontrovertible evidence, how often, on inquiry into the history of gouty invalids, they are found to have operated; and how, on physiological principles, those very symptoms which characterise the gouty diathesis naturally result from their operation. We shall very briefly refer to these debilitating causes.

"1. Years. After a certain period of mere existence, the brain loses its vital power, and cannot be restored; the susceptibility of the organ to usual stimulation and excitement is impaired; an imperfect and irregular exercise of the cerebral functions results; whence, in old people, mental hebetude and debility of body pervading all the senses and functions.

"2. The brain is often prematurely exhausted and impaired in its vital energies by various causes, so as to engender the gouty peculiarity.

"3. Excessive sensual indulgence is a most direct and influential means of wasting the vital power of the brain.

"4. Narcotic and spirituous liquors, habitually and largely used, also greatly impair the cerebral energy. How often this unfortunate habit of excess coexists with the gouty habit, needs no proof.

"5. Hard study, anxious excitement of mind, intense application, and night watching, do also wear out the cerebral power.

"6. The blood sent to the brain may be impure and imperfectly arterialised, and inadequate to maintain due vital energy of that organ.

"7. Mere deficiency in the quantity or nutritive quality of the blood, arising from excessive bleedings or impoverishing diet, directly weakens the powers of the brain.

"8. Any sedative, co-operating with the above causes, produces a much more decided effect on the cerebral power. In this way, cold applied to the surface of the body, from insufficiency in dress and exposure to a moist climate, habitually depresses the cerebral power.

"Such are the causes usually concerned in creating that depression and impairment of the cerebral power which ultimately terminates in the gouty state. Varieties, almost endless, occur, in the manner, degree, and complication in which their influence is exerted; some of which have been described in detail under the respective heads. Under all the circumstances and causes whose influence we have described, however, one uniform result has invariably been recognised,—i. e. a determination of blood to the head, supervening upon the derangement of the digestive functions, and the depression of the nervous energies, which paves the way for gout.

"From this general fact, therefore, we are entitled to infer that this determination of blood to the head has, in some way or other, an important, if not essential, connexion with the gouty state. In this inference we are strongly supported by the authority of Dr. Parry; that instructive writer having traced, through a number of gradations, those derangements of the circulation which precede the gout, and ultimately arrived at the conclusion that gout is an effort of nature to rectify a previous disordered state of the circulation, in which it is directed in excess to the alimentary canal and head. While we concur with this accurate observer in the matter of fact that a determination of blood to the alimentary canal and head does uniformly, in some degree, precede and coexist with the gouty peculiarity, we arrive at a different conclusion as to the manner in which the gout results from this pre-existing disordered state of these organs. We do not confound all impairment of the nervous system with a determination of blood to the brain, as that writer does. We do not blame nature in the business at all; but we state, as a practical observation, that when the nervous energies have been wasted, enfeebled, and impaired, by the various causes described in this treatise, a determination of blood to the brain is very liable to ensue upon any congestive condition of the organs of digestion, or indeed of any important vital organ. And when this determination of blood to the brain, in such a weakened state, has become habitual, the whole peculiarities of the gouty diathesis manifest themselves. The gout which ensues, so far from being a salutary effort of nature to relieve pre-existing disease, is itself a diseased result of the existing morbid state of the functions, in precise accordance with pathological laws." (P. 191.)

---

*Commentaries on the Causes, Forms, Symptoms, and Treatment, Moral and Medical, of Insanity.* By GEORGE MAN BURROWS, M.D. Member of the Royal College of Physicians of London, &c. &c.—8vo. pp. 716. Thos. and Geo. Underwood, London, 1828.

ALTHOUGH we may congratulate ourselves upon having, to a great extent, escaped from the thraldom of the mystified doctrines of the ancient writers upon the general subject of insanity, the purely practical inquirer still too frequently shrinks from this very important investigation, from an unfounded apprehension that he must dip deep into the perplexities of metaphysical disquisition, before he can gather any information upon the subject which will enable him to approach the treatment of mental diseases with any chance of success. Let us, then, premise our notice of the volume before us with an assertion which we do not believe can be refuted. Our treatment of mental diseases, as they are termed, will not be improved by abstract speculations as to the nature and seat of the mind, or intelligent soul. We know nothing of disease of the mind unconnected with corporeal ailment; and, whatever may be the kind or degree of the mental disturbance, we can only expect to be led to its relief by the same train of inquiry which is demanded in diseases which are unattended by any derangement of the manifestations of mind. It has been very justly concluded by PINEL, that all the speculative writings on the abstract essence of mind, and on the analysis of the human understanding, have contributed nothing to elucidate its disorders. Various hypotheses have been created for the purpose of explaining the nature and seat of the mind, or intelligent soul, and victory has been claimed by their propounders because their speculations have not been positively refuted. But where is the triumph? The fanciful doctrines to which we refer, like the arguments of PARACELSUS, still remain unanswered because they are unintelligible.

We are happy to find that Dr. BURROWS does not commit the much-to-be-lamented and frequent fault of encumbering a really difficult subject with either psychological or metaphysical abstrusities. In pursuing their inquiries into the nature of mind, the ancients plunged so deeply into the mysticism of metaphysics, that they lost sight of the true object of their research.

"The dogma that the soul, or mind, was a divine and divisible principle, governing and directing the intellectual faculties, but independent of organic matter, or (in other words) the body, fascinated and absorbed their whole attention. The opinions thence imbibed have descended through intervening ages, were revived with renewed ardour in the seventeenth and eighteenth centuries; and still, in the nineteenth, exercise a controlling influence.

"The effect has been to consider mental derangement not as a disease connected with the grosser or corporeal part of man, and within the province of medicine, but as a subject of abstract contemplation." (P. 3.)

CICERO, an acute observer, remarked that the nature of the human mind was too subtle for our weak perceptions to discover.* Of its disorders, "the absolute source, if even fully developed," says BACON, "will be found to exist in corporeal changes, or the effects of external agents acting on the gross machine, and not primarily on the immaterial principle, as has, unfortunately for the subjects of disease, been too commonly apprehended.†

Avoiding the dangerous, yet seductive, example of giving free scope to his imagination, by entering into abstract discussions as to the essence of mind, Dr. Burrows steers the more rational course of entering upon the consideration of questions which we trust do not mock the puny comprehension of man. "Let us be content," he wisely recommends, "instead of seeking its essence, to analyse the operations of

* Tusc. Disput. lib. i. † Novum Organon.

mind in health, and to endeavour to unfold the causes which influence those operations to the injury and derangement of its functions."

It has been imagined by some persons that our inquiries, in cases of insanity, should refer only to the mental symptoms, and that these are to be conducted in the same way as a clinical examination of the symptoms of bodily diseases. Having by this investigation determined the internal disorganization of the understanding whence the aberration originates, we may, they contend, trace it to its source, and ascertain the mental process by which it was formed. We fully agree with Dr. Burrows that they who argue thus know little of the matter. The attempt at treating an insane person in this manner would end in aggravation of the patient's state, and in certain disappointment and regret to themselves.

Dr. Burrowes conceives that the various phenomena which insanity presents have not been sufficiently studied, either in concurrence or sequence, or in relation to or combination with other cerebral affections. Impressed with this opinion, and that a more careful examination of the causes, both moral and physical, as well as of the various morbid affections in connexion with mental derangement, will lead to a clearer view of the pathology of insanity, he has entered into a wider field of investigation.

Commentary I. Moral Causes.—The substance of this introductory commentary may be comprised in a small space. Every impression on the sensorium, through the external senses, may become a moral cause of insanity. All impressions that affect the feelings are conveyed to the sensorium, and operate according to the degree of constitutional susceptibility and the nature and force of the impression.

"The action of the heart is correspondent with this impression, and reacts on the brain and nervous system. Hence there are two impressions: the one primitive, affecting the sensorium; the other consecutive, but simultaneously affecting the heart. Thus the nervous and vascular systems are both implicated; and in this manner moral impressions become causes of insanity. (P. 9.)

The effect of intense emotions or passions, often repeated or long continued, not only disturbs the functions, but will occasion lesions of the brain. Many structural and functional diseases, which are ascribed to physical causes only, may be clearly traced to emotions of the mind.

Mental derangement, it is well known, may ensue from the most opposite passions and sensorial impressions. "Joy and grief, anger and pain, love and hatred, courage and fear, temperance and ebriety, repletion and inanition, application and indolence, may have the same effect." We do not remember to have found the cardinal virtue of "temperance" ever before accused of leading to insanity.

Dr. Burrows records the following fact, of which we should have been doubtful, had it not been stated upon good authority:

"Actual losses, or disappointments in pecuniary speculations, do not appear to occasion insanity so frequently as unexpected or immense wealth. In the six months succeeding the extensive failures, and consequent distress, of the winter of 1825-6, in this metropolis, there were fewer returns of insane persons in the London district than in any corresponding period for many years past." (P. 16.)

Admitting, as every attentive observer must, the extensive influence of moral causes in the production of insanity, Dr. Burrows cannot assign it so wide a scope as many foreign writers have done. He entertains strong doubts of the fidelity of the catalogue of moral causes which they enumerate with so much affectation of minute accuracy. He has been very careful in his inquiries upon this point, in every case on which he has been consulted, and has "very frequently" been unable to trace any moral cause at all. "The majority originate in direct physical causes, which the privations, and consequent misery, the poor suffer in all countries, as well as their vices, greatly multiply."

Upon this subject the author appears to us to express himself too strongly. Granting that "no moral cause" can in some cases be traced, we at the same time doubt the *frequent* occurrence of such examples. It must evidently also be impossible to separate the moral from the physical effects induced by "privations, misery, and vice."

It is a mortifying yet undoubted fact, that, the higher the degree of civilization, the greater will be the tendency to insanity. We are hence led to exclaim, with ROUSSEAU, "tout est bien en sortant des mains de l'auteur des choses; tout dégénere entre les mains de l'homme." It must not, however, be consequently inferred that savage nations are exempt from this malady.

"The natives of the Indian peninsula, who are far more temperate in diet, and have their passions much more under control, are yet very prone to insanity; and several asylums are now established in the different presidencies for their reception. It is true they are more civilised than the American aborigines; but if civilization bring not with it the wants and vices, and consequent diseases, of Europeans, the exciting causes of mental derangement among the Peninsular Indians appear to be inadequate to produce this physical effect. As they are indubitably a very ancient race, hereditary predisposition probably exercises a considerable influence upon them." (P. 21.)

In every climate, and in every condition, man is so much the slave of his passions, as to be liable, among other ills, to madness.

Commentary II. ***Religion in reference to Insanity.***—We do not believe that religion, in the correct acceptation of the term, can ever be assigned as a cause of mental derangement. Religion is two-fold, true or false. True religion, in the language of the eccentric BURTON, "rears the dejected soul of man: it is a sole ease, an unspeakable comforte, a sweet reposal." False religion is either the vain superstition of idolatry, or the wanderings of a doubting mind, which can settle upon no fixed, rational, or consolatory belief. SENECA has wisely concluded, "*Religio Deum* colit, superstitio destruit." True religion, *ubi verus Deus vere colitur*, is the "mother of all virtues," and the soother, not the disturber, of the human mind. Dr. Burrows remarks, that "as there is no single passion, when excited to excess, that may not induce mental derangement, so we may readily believe that religion, which influences the internal man more than the passions collectively, may be a cause of insanity. On the other hand, there is no doubt that a lunatic may imbibe a religious as well as another hallucination, and yet be insane from a cause the reverse of religious. In the one case, however, it is a cause, in the other an effect." It would appear that in this passage the author refers to wild fanaticism, rather than to that pure and collected sense of piety which ensures a cheerful mind, which links the welfare of every particular with that of the whole, and to which only with correctness the term religion can be applied.

We are very properly guarded against the very common error of determining the moral cause of the malady from the tenor of the mental aberration. "It is to be feared that many cases have been hastily attributed to a religious origin, merely because the conduct or conversation of the lunatic has exhibited traits of too vivid spiritual impressions." Although Dr. Burrows is of opinion that, under certain circumstances, insanity is occasioned through the agency of religion, he explicitly declares that "it is not from the agency of the Christian faith, in its pure and intelligible form, but from the perversion of it, that many become the victims of insanity." There is no evidence to substantiate that Christianity abstractedly ever produced that effect. In England, where the mass of the people are piously and morally inclined, and where the liberty of theological discussion and religious worship is tolerated, every variety of schism and sectarism abounds. Numbers exchange one form of faith for another, and hence the work of proselytism is exceedingly prolific. This, it is very rationally contended by the author, "is the great predisposing cause to what is designated religious madness." The tenets entertained and promulgated may be highly dangerous to the happiness of proselytes, though innocuous to those bred in them.

"Error, till it be known to be such, bears the semblance of truth. Therefore, he who follows with sincerity that form of religion which he has been accustomed to consider as the true one, till he begin to doubt, is not likely to have either his conscience or understanding disturbed on that account. But if doubt arise, and he questions himself, or is questioned, on points of doctrine which he had cherished as orthodox, he may, in the misgivings which ensue, and in the uncertainty whether the old or new path be the right, unless he have a very strong mind, find himself in interminable perplexity. It is in this state the intellectual faculties are most apt to aberrate. The ideas then become fugacious, the conduct corresponds, and insanity is developed." (P. 32.)

Dr. Burrows does not, in fact, recollect an instance of insanity implying a religious source in any person stedfast to his ancient opinions.

A different view is taken of this question by an intelligent writer upon insanity. Dr. KNIGHT* has only once clearly ascertained, out of nearly seven hundred cases of insanity, that either a religious or a moral cause produced the disorder. He has uniformly found that the patient had betrayed at least equivocal symptoms of insanity before he became a raving devotee; and he contends that from this state of mind has arisen that proneness to change his mode of worship so frequently noticed in those who are termed religiously insane, and "*not* that the change in his mode of worship has caused the insanity."

Dr. Burrows gives several very interesting examples of insanity arising from perverted notions of religion, or from the admission of novel and controversial doctrines. The first is a melancholy instance.

"A single lady, about eight and thirty, enjoying good health, naturally of a cheerful temper, and regular in her devotions according to the rules of the established church, went, in the winter, on a visit. The family she visited were followers of Swedenborg. Partly through importunity, and partly from complaisance, she

* Observations upon the Causes, &c. of Derangement of the Mind. By PAUL SLADE KNIGHT, M.D. 1827.

attended their worship, and listened to the doctrines propounded. For the first time, perhaps, she catechised her present opinions; doubts arose; and, ere she had renounced her former belief or had adopted the new, she returned home to the vicinity of London. She shewed great and unusual disquietude of mind. Easter Sunday following, which was shortly after her return, she accompanied her mother to church. She stopt to receive the sacrament. There were many communicants; and, when the chalice was presented to her in turn, upon lifting it to her lips, she perceived that not a single drop of wine was left for her! She was excessively disconcerted and confused, hurried from the altar in dismay, and retired from the church. She declared she was lost; for the emptiness of the cup proved she was rejected of God! A furious paroxysm of mania ensued. It was, however, only temporary; and she in a short time regained her former composure.

"This lady soon after married, and was happy in the connexion: but has twice since, about Easter, when her mind has been naturally called to the religious duties of that period, fallen into a state of great despondency. She, however, has sustained the affliction of losing her beloved husband with all the fortitude and resignation of a true Christian.

"In this case, if the religious principles she had always professed had not been unsettled by the new doctrines she had heard, the casualty that proved the exciting cause of the maniacal paroxysm would have failed of any marked effect. (P. 40.)

Five out of the six examples related are females. The selection, the author observes, is not designed. He believes that nearly the same disproportion would be found between the sexes, if every case of insanity complicated with religion were recorded. The explanation of this fact is obvious. Man is physically more robust, and has less sensibility, or irritability, than woman. His education is more solid, and his mind more stedfast. More than one instance has fallen under our observation, in proof of the truth of the following observations.

"Were I to allege one cause which I thought was operating with more force than another to increase the victims of insanity, I should pronounce that it was the overweening zeal with which it is attempted to impress on youth the subtle distinctions of theology, and an unrelenting devotion to a dubious doctrine. I have seen so many melancholy cases of young and excellently disposed persons, of respectable families, deranged from either ill-suited or ill-timed religious communication, that I cannot avoid impugning such conduct as an infatuation, which, as long as persevered in, will be a fruitful source of moral evil. The old Romans knew human nature better: they had a law which forbad any person entering upon the sacerdotal office before the age of fifty. This was to prevent theological discussions before an age sufficiently mature was attained. If such studies were likely to disturb a Roman of mature age, we may judge the probable influence on a modern of fifteen or twenty. Seriously, this practice is an alarming error: it is growing to an excess fatal to the preservation of intellectual sanity, and in a manner especially dangerous to the rising generation." (P. 56.)

In our next Number we shall resume our analysis of this very interesting and practical work.

---

*Elements of Descriptive and Practical Anatomy, for the use of Students.* By JONAS QUAIN, A.B. M.B. Member of the Royal College of Surgeons, and one of the Lecturers on Anatomy in the Medical School, Aldersgate-street.

THE student who qualifies himself for the exercise of any department of medicine or surgery, has no ordinary merit. He has to contend not only against the limited means of acquiring a practical knowledge of anatomy, but even the time allotted for this purpose can only enable him, by great industry, to become acquainted with the more important parts concerned in surgical operations.

It cannot be denied that the greatest improvements in modern anatomy are to be ascribed to the continental anatomists, who, by combining this study with physiology and other sciences, have imparted to it the highest interest, and made it rank among the philosophical pursuits.

Physiology can scarcely be said to be taught in this country, when it is considered that the space of four months is only allowed for a course of lectures on anatomy, including physiology, pathology, and the operations of surgery! The works of BICHAT, MECKEL, &c. are now, however, duly appreciated; and let us hope that the increasing cultivation of anatomical knowledge will soon enable us to rival the more favored schools of anatomy of the continent.

The object of Mr. QUAIN's "Elements of Descriptive and Practical Anatomy" is to make the student acquainted with the present improved state of anatomy, according to the best authorities, but combined with that system of practical demonstration in which the English schools have excelled. It is, in short, a work for the dissecting room, as well as for reference; and the student who is anxious to pursue the subject beyond the directions of our present practical Manuals, will find this book an excellent guide.

The author states that his objects have been—

"To give a condensed and methodical description of the different structures and organs which enter into the composition of the human body.

"To point out the most convenient methods of conducting their anatomical examination.

"To indicate some of the more important practical applications that may be made of the facts disclosed to the student during the progress of his inquiries.

"And, finally, to present abridged summaries of the most instructive principles of general anatomy.

The descriptions are given with clearness and precision, and the histories attached to each operation cannot fail to impart additional interest to the inquiring student.

We can safely bear testimony to the accomplishment of the author's intentions; while the "Elements," in fact, prove a complete system of anatomy, worthy of the perusal of the older students.

